# Motion-based dynamic light delivery to minimize laser-related thermal damage while preserving photoacoustic image quality

**DOI:** 10.1117/1.JBO.30.5.056008

**Published:** 2025-05-14

**Authors:** Junior Arroyo, Jiaxin Zhang, Muyinatu A. Lediju Bell

**Affiliations:** a Johns Hopkins University, Department of Biomedical Engineering, Baltimore, Maryland, United States; b Johns Hopkins University, Department of Electrical and Computer Engineering, Baltimore, Maryland, United States; c Johns Hopkins University, Department of Computer Science, Baltimore, Maryland, United States

**Keywords:** photoacoustic imaging, light delivery, laser safety, microscopy, single-cell segmentation

## Abstract

**Significance:**

Photoacoustic imaging has the potential to be integrated into surgical guidance systems. However, biosafety from prolonged laser exposure can limit the maximization of signal-to-noise ratios. Although cooling strategies can potentially mitigate thermal impact, the associated adverse effects necessitate an alternative strategy.

**Aim:**

We introduce a dynamic light delivery strategy that displaces a light source in a controlled manner during photoacoustic imaging, which is expected to both minimize laser-related thermal damage and maintain the image quality achievable with stationary light delivery.

**Approach:**

Monte Carlo simulations were performed to determine the impact of light source displacement on local energy density. A dynamic light delivery device was designed, prototyped, and evaluated with an experimental phantom to determine image quality. To assess potential laser-related thermal damage, *in vivo* swine liver was exposed to laser light delivered with 750-nm wavelength, nanosecond pulses, and 32.4 mJ median pulse-to-pulse energy for 20-min total duration, under both stationary and dynamic light delivery. The exposed liver samples were excised, followed by categorical grading and quantitative depth measurements of resulting hemorrhage observed in H&E liver sections.

**Results:**

Energy densities at the simulated tissue surface were 1.85 lower with dynamic rather than stationary light delivery. As target depth was varied from 14 to 53 mm, the median signal-to-noise and generalized contrast-to-noise ratios ranged 24.60 to 38.76 and 0.96 to 1.00, respectively, with stationary light delivery and 23.06 to 37.47 and 0.96 to 1.00, respectively, with dynamic light delivery, with no statistically significant differences between light delivery approaches (p>0.05). Histopathology of excised liver samples revealed mild hemorrhage with stationary light delivery that was reduced to minimal hemorrhage with dynamic light delivery, quantified as median hemorrhage depths reduced from 0.79 to 0.16 mm (i.e., 80% hemorrhage depth reduction).

**Conclusions:**

Dynamic light delivery is a promising approach to mitigate potential laser-related damage.

## 1 Introduction

Photoacoustic imaging has consistently showcased its ability to provide significant physiological insight from biological tissue in preclinical settings.^1^^–^^3^ This progress promises new possibilities to integrate photoacoustic technology into surgical guidance systems to enhance intraoperative decisions.^4^ Nanosecond pulses of laser light exposed to photoabsorbers within tissue cause local optical absorption followed by milliKelvin temperature increases that trigger thermal expansion followed by contraction. Light delivery to surgical sites can be achieved by coupling fiber optics to ultrasound receivers,^5^ positioning light at fixed distances from the receiver,^6^ or attaching optical fibers to surgical tools.^7^^,^^8^ The common feature among these approaches is a fixed light source (relative to the ultrasound receiver or surgical tool), resulting in what we refer to as stationary light delivery. A handheld light source can also be subtly operated within a narrow surgical window, which is also considered “stationary” light delivery (relative to other components that experience more motion).

Despite the promise of photoacoustic imaging with stationary light delivery, concerns surrounding potential thermal damage to biological tissues from high-energy laser sources^9^^,^^10^ limit the widespread clinical implementation of this imaging modality.^11^ To provide examples of what may be considered problematic (in the absence of updated safety guidelines), cardiac catheter-based interventions^12^ utilized $379.2\;\;\mathrm{mJ}/{\mathrm{cm}}^{2}$ fluence, and blood vessel visualization in the pancreas^13^ utilized $183.4\;\mathrm{mJ}/{\mathrm{cm}}^{2}$ fluence. When visualizing major hepatic vessels under likely surgical usage durations (e.g., up to 20 min),^13^^,^^14^ $152.6\;\mathrm{mJ}/{\mathrm{cm}}^{2}$ fluence was utilized. More recently, additional laser safety assessments revealed that liver tissue can tolerate up to $374.8\;\mathrm{mJ}/{\mathrm{cm}}^{2}$ over a 1-min duration.^10^ In each of these examples, the delivered fluence was considered safe (i.e., no laser-related tissue damage was observed), although the reported safety limit for skin (i.e., $25.2\;\mathrm{mJ}/{\mathrm{cm}}^{2}$ with the same 750-nm wavelength and 5-ns pulses used for these demonstrations^15^) is otherwise the current standard. If existing standards and guidelines remain slow to update, the required fluence can potentially be lowered within existing safety guidelines through novel technology designs.

There are additionally cases where the required energy is actually unsafe. For example, a targeted biological photoabsorber can potentially undergo prolonged exposure to light pulses, leading to undesirable effects, such as hyperthermia, rupture of molecular bonds, decreased enzyme activity, and protein or collagen denaturation.^10^^,^^16^ Tissue coagulation, cell necrosis, increased cell membrane permeability, and ablation are also possible.^10^^,^^16^

Multiple intraoperative strategies are currently employed to control thermal damage from laser exposure, including adjustments to laser parameters (e.g., wavelength, fluence, number of pulses)^17^ and the utilization of dynamic cooling devices.^18^ However, adjusting laser parameters can compromise light penetration, reduce contrast between chromophore absorption, prevent visualization of photoacoustic targets, or limit exposure duration (which is ultimately disadvantageous for photoacoustic-guided surgeries). While primarily used in dermatological applications, dynamic cooling devices (e.g., cold liquid,^19^ cold air,^20^ cooled balloons,^21^ cryogen spray^22^) can be adapted for use in other tissues to mitigate tissue overheating, enabling the application of increased laser energy and fluence. Disadvantages include vasoconstriction,^23^ the formation of an ice layer in the tissue surface, accumulation of cryogenic liquid, potential inhalation hazard in non-ventilated areas, and discomfort. Cold air devices in particular can be bulky and difficult to effectively manipulate, requiring two hands to operate.^24^ More generally, dynamic cooling devices are often restricted to use with specific laser systems, constraining their versatility.^24^

One potential alternative to address the limitations of dynamic cooling devices is to dynamically distribute light at a fixed rate of motion. A numerical study investigating the tradeoff between image quality and the radii of circular motion patterns revealed that continuous displacement of an optical source center along a 1.5-mm-radius trajectory negligibly reduces signal-to-noise ratios (SNRs) and offers the highest possible generalized contrast-to-noise ratio (gCNR),^25^ relative to stationary light delivery. These results provide initial parameters for a motion-based light delivery design that yields sufficient target detectability. Therefore, we hypothesize that with no changes to the beam energy or fluence, introducing a dynamic light delivery approach with the light source in continuous motion could potentially lead to a decrease in local energy density, resulting in a local temperature reduction and consequently minimizing thermal damage.

In this paper, we empirically investigate the effectiveness of light source motion to minimize thermal damage while preserving image quality during the photoacoustic imaging process, resulting in three novel contributions. First, we introduce an innovative device and its associated design to autonomously displace a light source to achieve dynamic light delivery. Second, we present a novel 14-step analysis framework to quantitatively estimate the maximum depth at which hemorrhage was observed from the irradiated surface in hematoxylin and eosin (H&E) digitized sections. Finally, we deploy the novel device in experimental phantom and *in vivo* settings to investigate the resulting image quality and thermal damage, respectively.

## 2 Methods and Materials

### 2.1 Simulations to Assess the Impact of Light Source Displacement on Energy Density

**Fig. 1:**
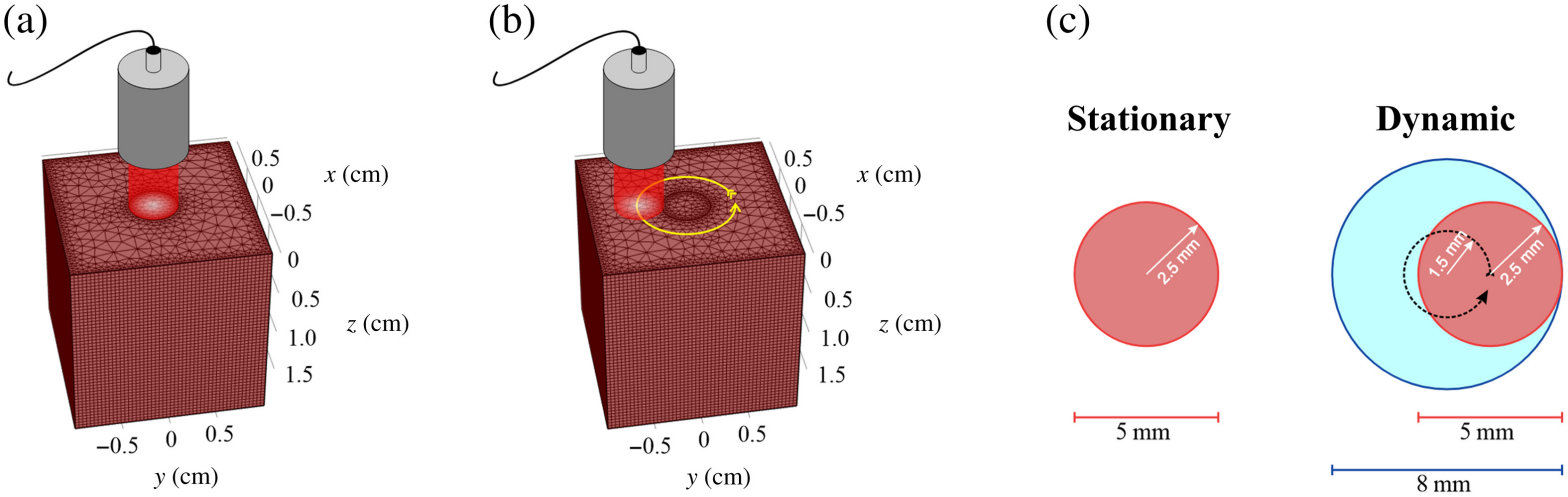
Porcine liver tissue model used for Monte Carlo simulations, with (a) fixed laser source positioned at the center of the exposed surface for stationary light delivery and (b) laser source displaced along a circular trajectory at the exposed surface for dynamic light delivery. (c) Comparisons of illuminated areas in contact with stationary (red) and dynamic (blue) light delivery.

To evaluate the impact of light source displacement on local energy density, 3D Monte Carlo simulations^26^ were conducted using a $2\times 2\times 2\;{\mathrm{cm}}^{3}$ homogeneous block of tissue (shown in Fig. 1). This tissue was modeled with the optical parameters of a vascularized swine liver specified in Table 1. A 5-mm diameter laser source was in direct contact with the top surface of the tissue, delivering pulses of 32.4 mJ energy with 750 nm laser wavelength, 5 ns pulse duration, and 10 Hz pulse repetition frequency.

**Table 1:** Optical parameters utilized in Monte Carlo simulations. These values were obtained from Ritz et al.^27^

Parameter	Value
Absorption coefficient	$0.10\;{\mathrm{mm}}^{-1}$
Scattering coefficient	$6.14\;{\mathrm{mm}}^{-1}$
Anisotropy factor	0.90

**Fig. 2:**
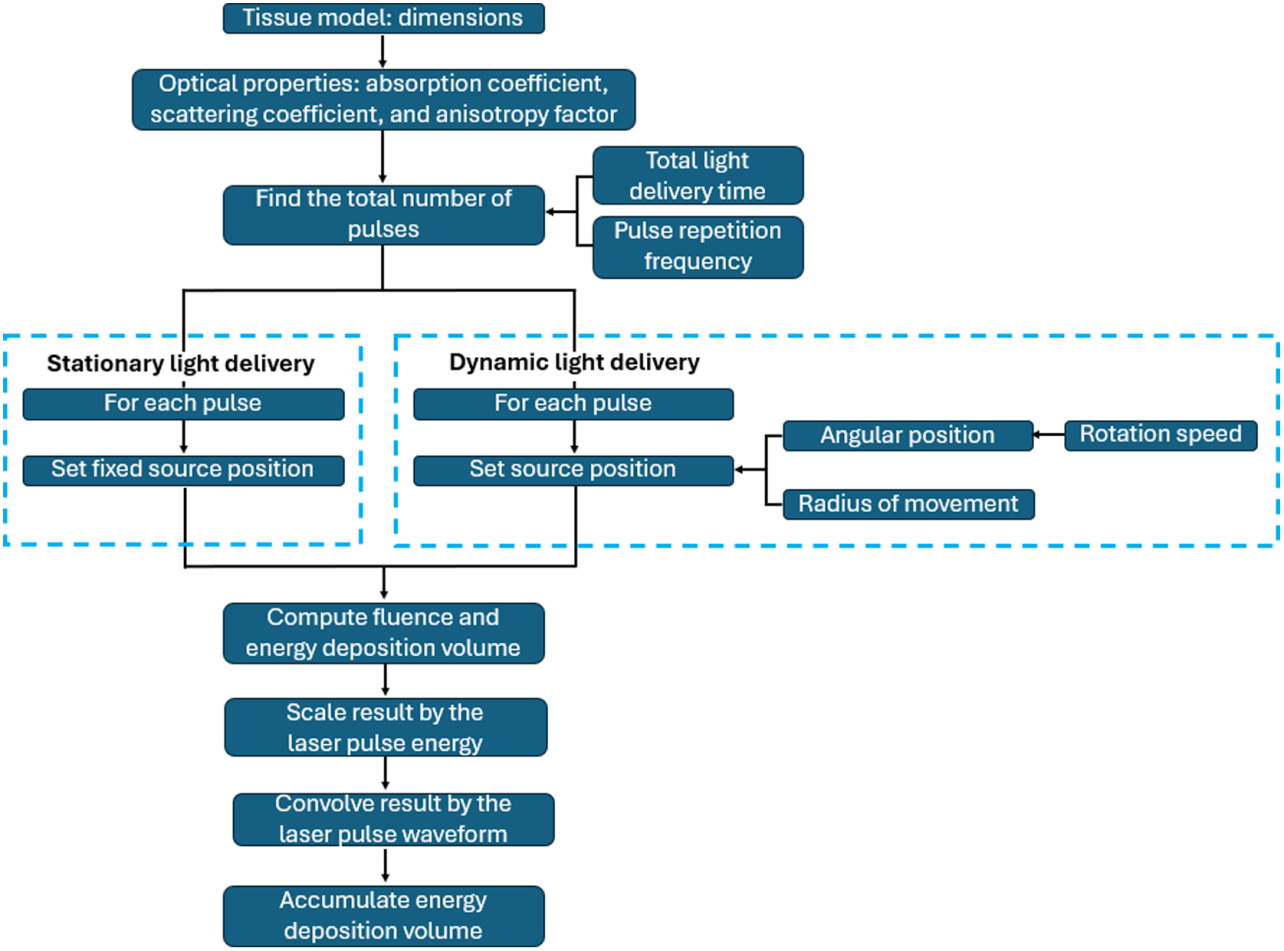
Monte Carlo simulation flowchart for stationary and dynamic light delivery.

To simulate stationary light delivery, the light source was positioned at the center of the exposed surface, and energy was delivered for 3 min. To simulate dynamic light delivery, a light source displaced with an angular speed of five revolutions per minute (RPM) was simulated following four steps. First, the total light delivery time was defined (i.e., 3-min exposure) and the number of pulses to be delivered within the elapsed time was determined. Second, the light source period and the number of pulses per period were calculated based on the selected angular speed. Third, the angular spacing between subsequent pulses was computed. Finally, the delivery position for each pulse was determined and the light propagation for each pulse was simulated. The simulation outputs of the 3D Monte Carlo were normalized cumulative energy density after 3-min laser exposure, which provided information about the spatial distribution of the deposited energy. To incorporate pulse energy, the initial simulation output was scaled by 32.4 mJ. To incorporate the temporal pulse waveform (i.e., a unit step function with 5 ns duration), this waveform was convolved with each voxel of the solution. Figure 2 summarizes the Monte Carlo simulation processes with stationary and dynamic light delivery.

### 2.2 Photoacoustic Imaging System

Our experimental photoacoustic imaging system consisted of an Alpinion ECUBE 12R ultrasound scanner (Seoul, Korea) synchronized with a Phocus Mobile laser (OPOTEK, Carlsbad, California, United States). The ultrasound scanner was connected to a 128-element Alpinion L3-8 linear array transducer (5 MHz center frequency, 3 to 8 MHz bandwidth, 0.3 mm pitch, 40 MHz sampling frequency, 64-element receive aperture). The laser was connected to a 5-mm-diameter fiber bundle, which delivered pulsed light with 750 nm wavelength, 5 ns pulse duration, and 10 Hz pulse repetition frequency.

### 2.3 Autonomous Light Source Displacement Device

**Fig. 3:**
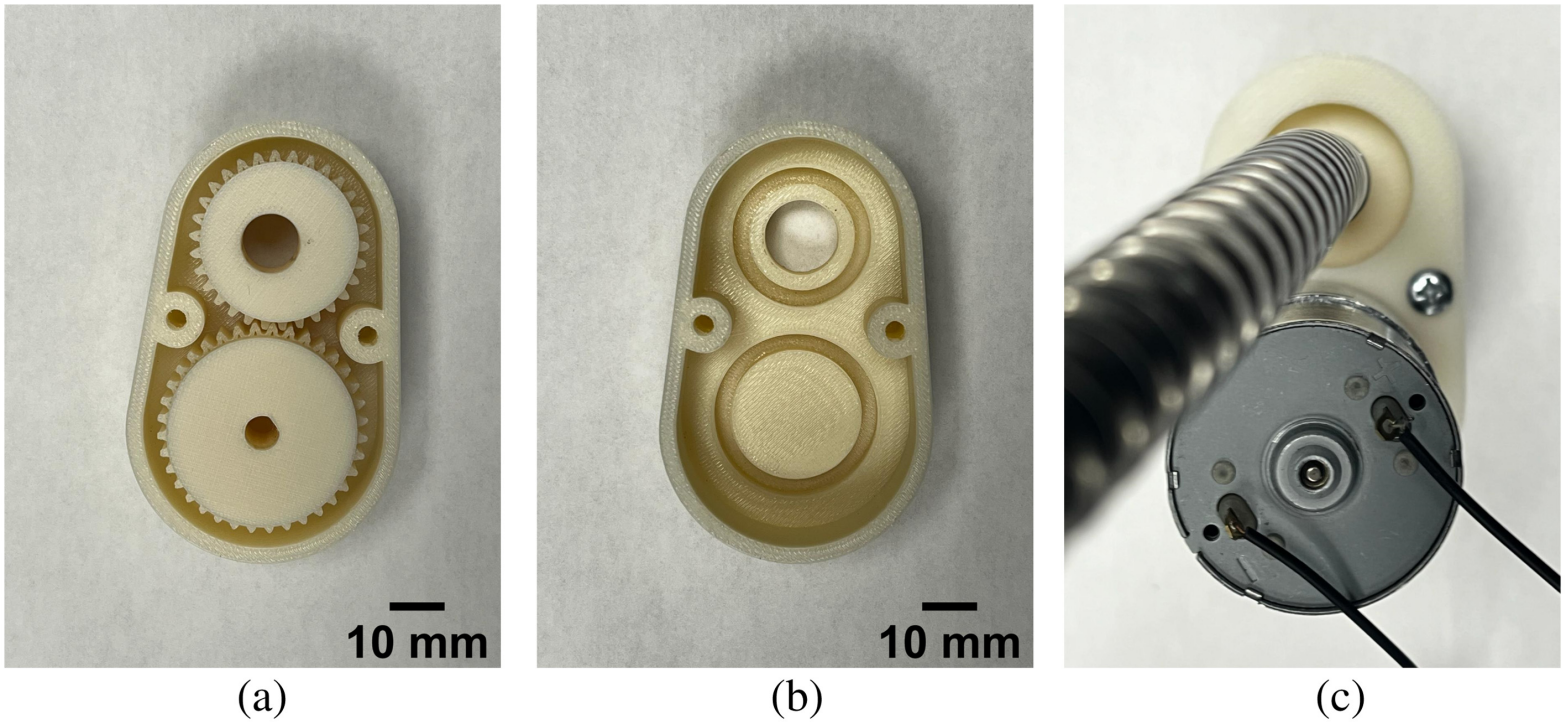
Laser positioning device with (a) geared mechanism for motion transfer, (b) a lower component with grooves for gear positioning, and (c) an upper component that stabilizes the motor and facilitates the positioning of the optical fiber bundle.

To administer the proposed motion-based dynamic light delivery approach, the optical fiber bundle described in Sec. 2.2 was mounted on a laser positioning device, which placed the light source in direct contact with the surface to be imaged and enabled the delivery of a ring-shaped light path at a constant angular speed. The mechanical device was designed to consist of two interconnected gears, as shown in Fig. 3(a), arranged to efficiently transfer motion between gears. The larger 33.4-mm-diameter gear was driven by the rotational force of a direct current (DC), 12 V, 20 RPM gear motor (Greartisan, China), which initiates motion within the dynamic system. This rotational motion is then transmitted to the smaller 27-mm-diameter gear, which contains a 5-mm-diameter orifice to accommodate a light source. The center of the orifice is offset by 1.5 mm from the center of the gear. This off-centered orifice generates a circular light source motion (with a 1.5-mm radius) as the gears rotate.

A 3D-printed housing was engineered to enclose the geared mechanism, comprising lower [Fig. 3(b)] and upper [Fig. 3(c)] halves fastened together with screws. The lower half of the housing [Fig. 3(b)] contains strategically positioned grooves to align the gears, enabling smooth gear rotation within the device. The upper half [Fig. 3(c)] accommodates the connection between the motor and gear mechanism. The upper half of the housing also supports and stabilizes the motor, preventing its weight from affecting gear motion. The remaining components include a universal transformer and a pulse width modulation (PWM) digital motor speed controller. The universal transformer was used to step down the voltage from the 120 V power source (i.e., a standard 120 V electrical outlet) to the 12 V required by the DC motor specifications. The PWM digital motor speed controller was integrated into the circuit to reduce the angular speed of the DC motor to 5 RPM.

### 2.4 Beam Profile Characterization

**Fig. 4:**
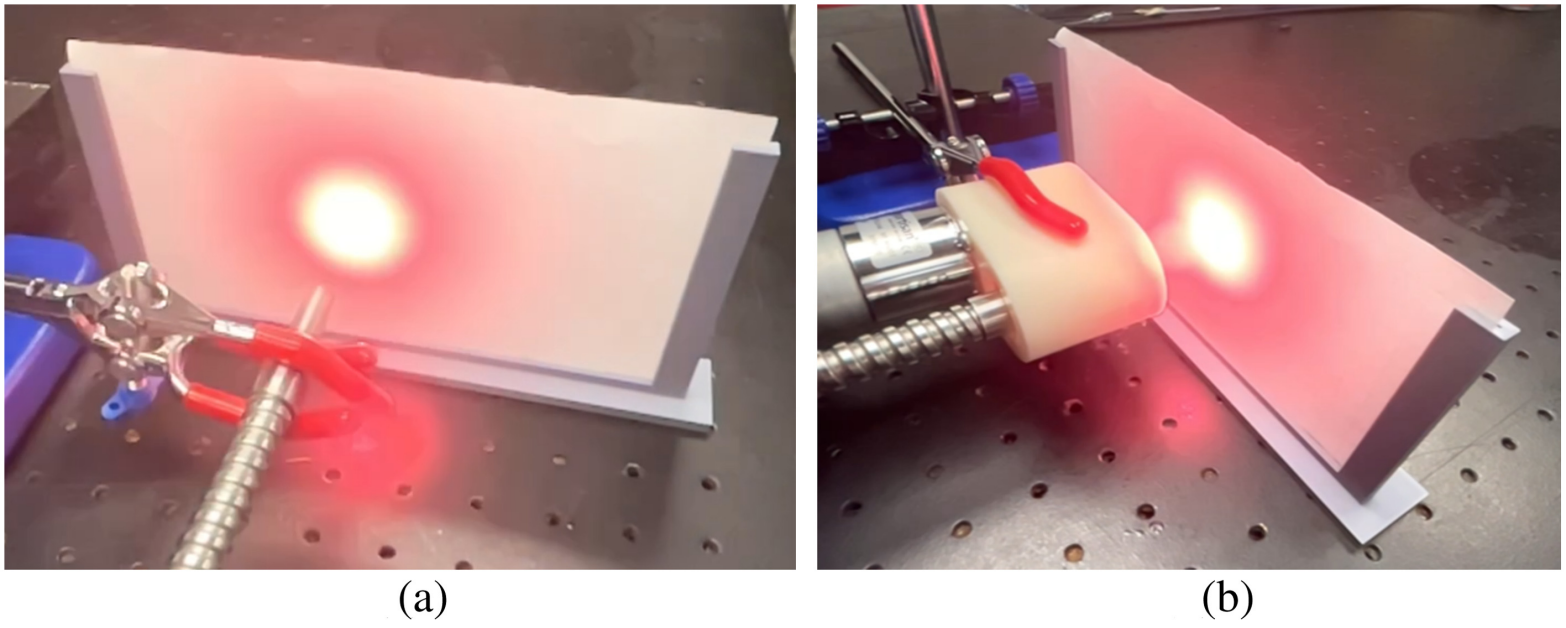
Experimental setup to characterize (a) stationary and (b) dynamic beam profiles.

To characterize beam profiles, the stationary or dynamic optical source was directed onto a sheet of grid paper, under similar room illumination conditions, as shown in Fig. 4. The mean ± one standard deviation laser pulse energy was 31.12±0.68  mJ, measured over 120 s (i.e., 1200 pulses). The light source was positioned at a distance of 31 mm from the paper grid. A single photograph of the illuminated grid paper was taken with the stationary light delivery approach. With dynamic light delivery, the light source was continuously rotated at 5 RPM, and photographs were captured at 1 s intervals over a single revolution (i.e., 12 s total). The resulting images were spatially aligned as a function of time to form a 3D tensor using MATLAB software. The maximum intensity along the time axis was projected as a function of spatial position, generating a single image representing the dynamic beam profile. Otsu’s method^28^ was applied to threshold the images obtained with stationary and dynamic light delivery, segmenting the photographed optical profile from the background. ImageJ was used to measure the radii of the segmented optical profiles.

### 2.5 Phantom Experiment to Compare Image Quality

**Fig. 5:**
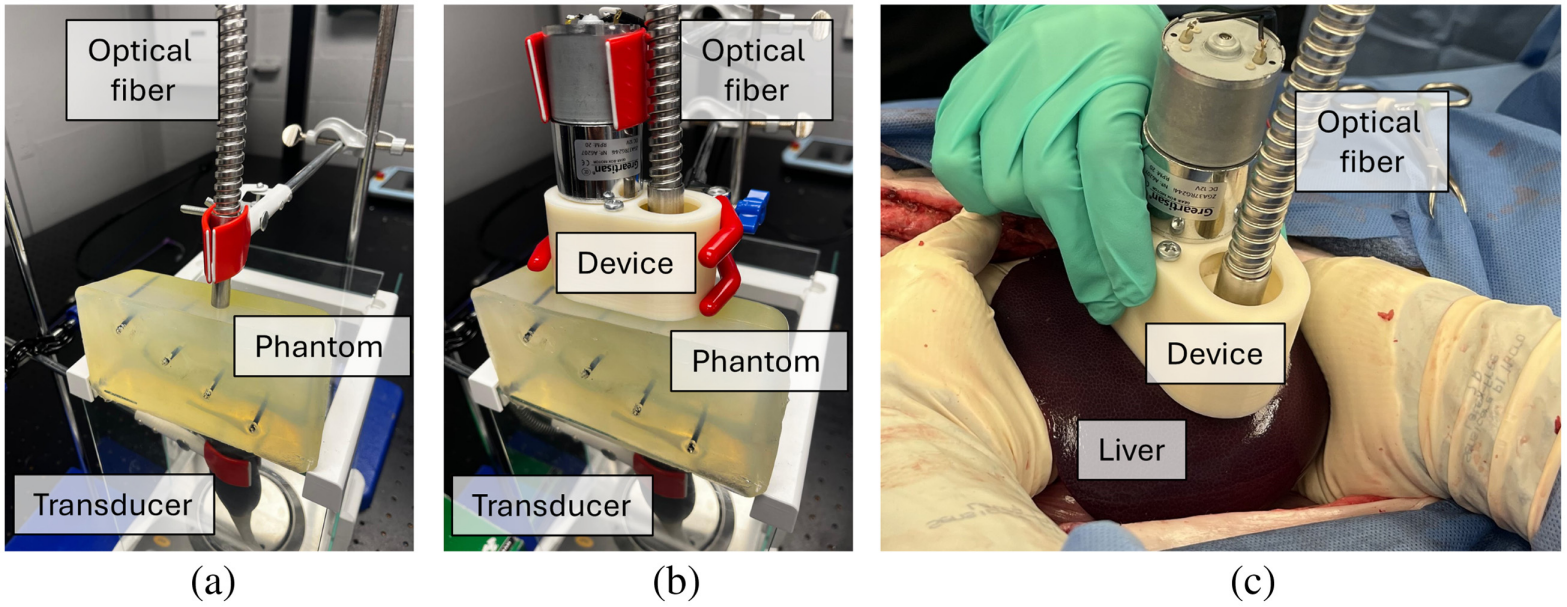
Experimental setups for (a) stationary and (b) dynamic light delivery applied to a phantom and (c) dynamic light delivery for thermal damage assessment of *in vivo* swine liver tissue.

A custom cubic polyvinyl chloride-plastisol (PVCP) phantom containing four 3-mm-diameter ink photoacoustic targets (Dr. Ph. Martin’s Bombay India Inks, Salis International, Inc., Golden, CO, United States) was fabricated to determine the impact of the light delivery method and target depth on image quality. Targets were positioned at depths of 14 mm, 26 mm, 39 mm, and 53 mm. Each light delivery method was placed on one surface of the phantom, as shown in Figs. 5(a) and 5(b), and the ultrasound transducer was placed on the opposite surface, laterally aligned with the optical fiber. The mean laser energy measured at the optical fiber tip was 32.1 mJ per pulse (median: 32.2 mJ). The dynamic light delivery rate was initially 5 RPM. There were a total of 24 photoacoustic image acquisitions (i.e., 3-min total acquisition duration) per light delivery method per target depth illumination.

To determine the impact of rotation speed on image quality, the original motor of the laser positioning device was replaced with a DC, 12 V, 100 RPM gear motor (Greartisan, China). This replacement allowed us to test the following five additional rotation speeds when imaging the target located at 26-mm depth: 20, 40, 60, 80, and 100 RPM. There were a total of 24 photoacoustic image acquisitions (i.e., 3-min total acquisition duration) per rotation speed.

To assess image quality, photoacoustic images were first reconstructed from the acquired channel data using delay-and-sum beamforming with an assumed sound speed of 1400  m/s.^29^ The SNR and gCNR were calculated using the following equations:^30^^–^^32^

$$\mathrm{SNR}=\frac{\mu_{t}}{\sigma_{b}},$$ (1)

$$\mathrm{gCNR}=1-\overset{N}{\underset{k=1}{\sum }}\;\min\{h_{t}(x_{k}),h_{b}(x_{k})\},$$ (2)

where $\mu_{t}$ is the mean, $\sigma_{b}$ is the standard deviation, and $h_{t}$ and $h_{b}$ are the histograms of the signal amplitudes (after envelope detection, scan conversion, and normalization) within circular (i.e., 0.9 mm radius) regions of interest (ROIs) placed at the same image depth within the photoacoustic target (denoted by subscript t) or within the background of the photoacoustic target (denoted by subscript b), N=256 histogram bins, and $x_{k}$ is the mean value of the k’th bin. The target ROI was manually positioned inside and centered on the target. The background ROI was laterally displaced by 2.5 mm from the target ROI. When displaying boxplots to represent image quality results, the horizontal line within and the height of each box represent the median and interquartile range, respectively. The vertical lines above and below each box extend to the maximum and minimum values, excluding outliers, which are defined as values exceeding 1.5 times the interquartile range.

### 2.6 *In Vivo* Swine Experiment to Assess Thermal Damage

#### 2.6.1 Laparotomy

The following *in vivo* procedures were approved by the Johns Hopkins University Institutional Animal Care and Use Committee. A laparotomy was performed on the abdomen of a female Yorkshire swine (36 to 40 kg) to expose the left medial liver lobe, as shown in Fig. 5(c). To monitor and compensate for known fluctuations in energy and maintain a desired sliding-average pulse-to-pulse energy range over any group of 50 consecutive pulses, the internal power meter of the laser and a custom command-line interface were employed.^14^ In particular, a calibration between the internal and external power meters was performed prior to the procedure. The internal power meter was then used to record and adjust real-time laser energies throughout the procedure. For each light delivery method, the interquartile energy range was 30 to 33 mJ per pulse with a median value of 32.4 mJ. The mean energy values were 32.3 mJ per pulse and 32.4 mJ per pulse with stationary and dynamic light delivery, respectively.

Suture lines perpendicular to and 3 cm away from each intended laser application site were placed on the left medial lobe. Stationary light delivery was applied to a single position on the caudal surface of the lobe for a duration of 20 min. Dynamic light delivery with an angular speed of 5 RPM was applied on the cranial surface of the lobe for a duration of 20 min. Tissue marking dye was then applied to the irradiated regions, using the sutures as a guide. The entire left medial liver lobe was then removed from the abdomen after euthanasia. The marked irradiated regions were subsequently excised and immediately fixed in a 10% formalin solution.

#### 2.6.2 Histopathology

To analyze the depth of potential light exposure effects and evaluate the local impact of laser exposure from each light delivery approach, the two formalin-fixed liver samples described in Sec. 2.6.1 were embedded in paraffin and placed in a microtome with the irradiated surface oriented approximately perpendicular to the microtome blade. Each sample was continuously sectioned with a thickness of 40  μm, resulting in 142 unstained sections from the stationary light delivery and 199 unstained sections from the dynamic light delivery. Sections separated by 200  μm were selected for staining with H&E, resulting in 29 and 40 stained sections from the stationary and dynamic light delivery approaches, respectively. However, due to the irregular shape at the edge of the tissue as a consequence of manual resection, fragmented areas were included in five stained sections obtained per light delivery method. To minimize the out-of-distribution assessment of depth-dependent thermal effects, these sections were excluded from depth analyses. Therefore, only the remaining 24 and 35 H&E-stained sections corresponding to stationary and dynamic light delivery, respectively, were included in the following analyses.

Individual H&E sections were digitized at 40× magnification using a Hamamatsu NanoZoomer S210 to generate NDPI files. The NDPITools Plugin Bundle of ImageJ was used to extract the content of the NDPI file by dividing each digitized H&E section into a mosaic of adjacent JPEG images. When these multiple JPEG images were spatially aligned and stitched together, the H&E section was accurately reconstructed. Pathological features (i.e., hemorrhage, inflammation, degeneration, and necrosis) were qualitatively assessed from the H&E stained sections. Tissue conditions were categorized into five levels of damage: not present, minimal, mild, moderate, and severe. Hemorrhage is particularly of interest due to its presence in previous *in vivo* swine liver specimens exposed to similar laser energies and time durations.^13^^,^^14^

#### 2.6.3 Hemorrhage depth measurements

To quantify differences in thermal impact between stationary and dynamic light delivery, we measured the maximum depth at which hemorrhage was observed from the irradiated surface (i.e., hemorrhage depth). The hemorrhage depth measurement process consisted of 14 steps: (1) segmentation of red cells from individual JPEG images, (2) formation of a hemorrhage map using the segmentation masks of individual JPEG images, (3) rigid registration between H&E sections, (4) rigid registration of hemorrhage maps, (5) extraction of the tissue boundary, (6) ROI selection from the H&E section to depict the irradiated area, (7) ROI generation from the hemorrhage map, (8) identification of the irradiated boundary, (9) two-stage filtering procedure to remove artifacts from the ROI of the hemorrhage map, (10) linear fitting of the irradiated boundary, (11) extraction of relevant points from the linear-fitted irradiated boundary, (12) projection of the relevant points to the non-fitted irradiated boundary, (13) distance calculations between red cells and the projected relevant points, and (14) calculation of hemorrhage depth in millimeters. Additional details about each of these steps are provided below, which were applied to each H&E image and corresponding hemorrhage map from the dataset detailed in Sec. 2.6.2. Segmentation (Step 1) was performed using Python 3.9.15 in a Jupyter Notebook, whereas the remaining image processing steps (Steps 2 to 14) were performed using MATLAB R2023a (Mathworks, Natick, MA, United States) software.

Step 1: The segmentation of red cells was performed using an Attention U-Net,^33^ which consisted of four encoder layers, four decoder layers, and four attention gates. This network was trained using the Adam optimizer, a batch size of four samples, and standard data augmentation techniques (i.e., flip, elastic transformation, grid distortion, and optical distortion). Early stopping with a patience of 10 was employed to avoid overfitting. The Attention U-Net was trained with JPEG images from the current dataset. The network dataset contained 50 images along with their manual segmentations, with an 80%-20% training-testing set split, and 20% of the training set formed the validation set. The performance of the segmentation was quantified using the dice similarity coefficient (DSC),^34^ intersection over union (IoU), recall, and precision. The weights obtained from this training process were stored and utilized for cell segmentation in individual JPEG images.
Step 2: The segmentation masks of red cells in individual JPEG images were spatially aligned and stitched together to generate a segmentation mask of the entire H&E section, which we define as a hemorrhage map.
Step 3: Rigid registration was performed to align individual 2D serial digitized H&E sections into a common coordinate frame. A section from the middle of the H&E stack was selected as the common frame to avoid reconstruction artifacts or helical volumes.^35^ The registration was performed in forward and backward directions (i.e., pairwise registration until the last and first sections, respectively) using the MATLAB function imregtform, with an initial radius of $6.25\times 10^{-4}$, a maximum iteration number of 800, and four pyramid levels. Each H&E section was converted from the RGB to $L^{*}a^{*}b$ color, using only the $a^{*}$ (red/green) coordinate to enhance tissue visualization and aid in automatic feature extraction.
Step 4: Each pair of adjacent H&E sections evaluated produced a deformation map, which was then used to warp the corresponding hemorrhage maps. This step placed these maps into a common coordinate frame to facilitate the identification of the irradiated boundary in each H&E section.
Step 5: A mask delineating the tissue boundary was generated by transforming the warped H&E sections into grayscale images, then computing a morphological image, generating a binary mask using a threshold value of 0.03, and finally applying the MATLAB function edge using the zero-crossing method.
Step 6: To optimize runtime, the analysis was restricted to the irradiated area rather than the entire tissue section. Therefore, three ROIs were generated. An ROI of the H&E section was manually selected and cropped, and the ROI coordinates were stored.
Steps 7 and 8: Using the stored ROI coordinates, ROIs from the hemorrhage map (Step 7) and the tissue boundary image (Step 8) were obtained, depicting exclusively the irradiated boundary and the underlying red cells.
Step 9: A visual inspection of the ROI from each H&E section revealed the presence of features not related to thermal injury (e.g., scattered individual red cells, aggregates of few red cells, vessels), which may negatively influence the hemorrhage depth measurement. In the ROI of the hemorrhage maps, the minor axis length of a single red cell was ∼10  pixels, aggregates overall comprised less than 300 red cells, and vessels were characterized as large circular to oval entities packed with red cells. Therefore, a two-stage filtering process was implemented to eliminate these features while retaining hemorrhage-related clusters of red cells. In the first stage, a morphological dilation operation was applied to the ROI of the hemorrhage map, using a disk with a radius of 5 as a structural element. Subsequently, binary objects smaller than 3000 pixels were removed, filtering out individual red cells and aggregates. The output of this stage is referred to as a preliminary filtered hemorrhage map. In the second stage, two sets of coordinates were obtained. The first set of coordinates corresponded to all pixels forming the irradiated boundary, whereas the second set corresponded to the centroid of all hemorrhage clusters from the preliminary filtered hemorrhage map. The shortest distance between a hemorrhage cluster and the irradiated boundary was then calculated. Clusters placed less than 600 pixels of distance below the irradiated surface were most likely to be hemorrhage-related, as found by inspection of the hemorrhage maps from our dataset. Therefore, any cluster for which the distance exceeded the empirical value of 600 pixels was classified as an artifact and consequently removed. The output of this two-stage filtering procedure is referred to as a filtered hemorrhage map.
Steps 10–13: To address boundary irregularities, linear fitting was performed on the irradiated boundary (Step 10). A perpendicular line to the fitted boundary that intersects each red cell from the filtered hemorrhage map was calculated to extract relevant points on the fitted boundary (Step 11). These boundary points were projected onto the non-linear irradiated boundary (Step 12), and the distance between the projected points and each red cell was computed (Step 13).
Step 14: The largest computed distance was then retained and expressed in millimeters.

### 2.7 Statistical Analyses

The following statistical analyses were applied to image quality measurements (Sec. 2.5) and hemorrhage depth measurements (Sec. 2.6.3). First, the Shapiro-Wilk test determined the normality of each dataset distribution, and Levene’s test evaluated variance equality. The Mann-Whitney U test was then used to determine statistically significant differences between light delivery methods based on image quality metrics and hemorrhage depth measurements. These statistical analyses were conducted at the 0.05 significance level, using Python 3.9.15.

## 3 Results

### 3.1 Effect of Light Source Displacement on Energy Density

**Fig. 6:**
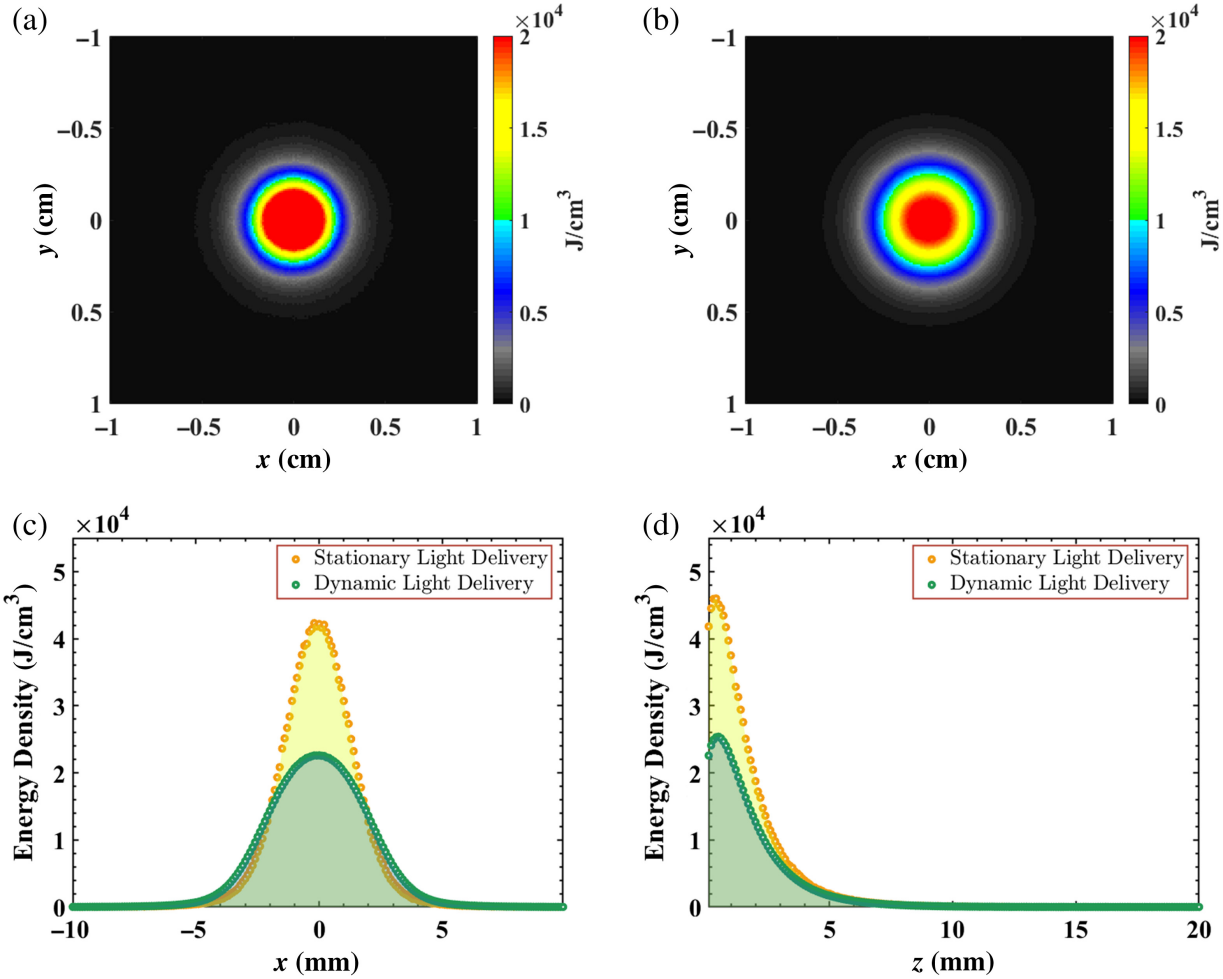
Energy density maps at z=0  cm (i.e., at the laser-tissue interface) obtained with (a) stationary and (b) dynamic light delivery. Corresponding profiles of energy density (c) as a function of x at the tissue model surface (y,z)=(0,0)  cm and (d) as a function of depth, normal to the tissue surface at (x,y)=(0,0)  cm.

Figure 6 shows the simulation results of the measured energy densities. In Figs. 6(a) and 6(b), maps of the energy density at the interface between the laser and tissue model (i.e., z=0  cm) are displayed, achieved with stationary and dynamic light delivery, respectively. A larger area of tissue was illuminated when the light source was displaced (i.e., $0.50\;{\mathrm{cm}}^{2}$), compared with that of the stationary light source (i.e., $0.19\;{\mathrm{cm}}^{2}$), which is consistent with the diagram in Fig. 1(c). Corresponding line plots of energy density along the tissue surface [Fig. 6(c)] and as a function of depth into the tissue [Fig. 6(d)] are also shown. In Fig. 6(c), a lower energy density was achieved with the displaced (rather than stationary) light source. In Fig. 6(d), the two laser delivery methods penetrated to the same tissue depth of ∼8  mm, but the local energy density differed. In particular, at the exposed tissue surface, the energy density from stationary light delivery was 1.85 times greater than that of dynamic light delivery. The energy density at 3 mm from the surface increased due to scattering and reflection interactions between the delivered photons and the exposed tissue, which has also been observed in previous work.^36^ As depth from the tissue surface further increased, the energy density decreased to zero.

### 3.2 Effect of Light Delivery on Beam Profiles

**Fig. 7:**
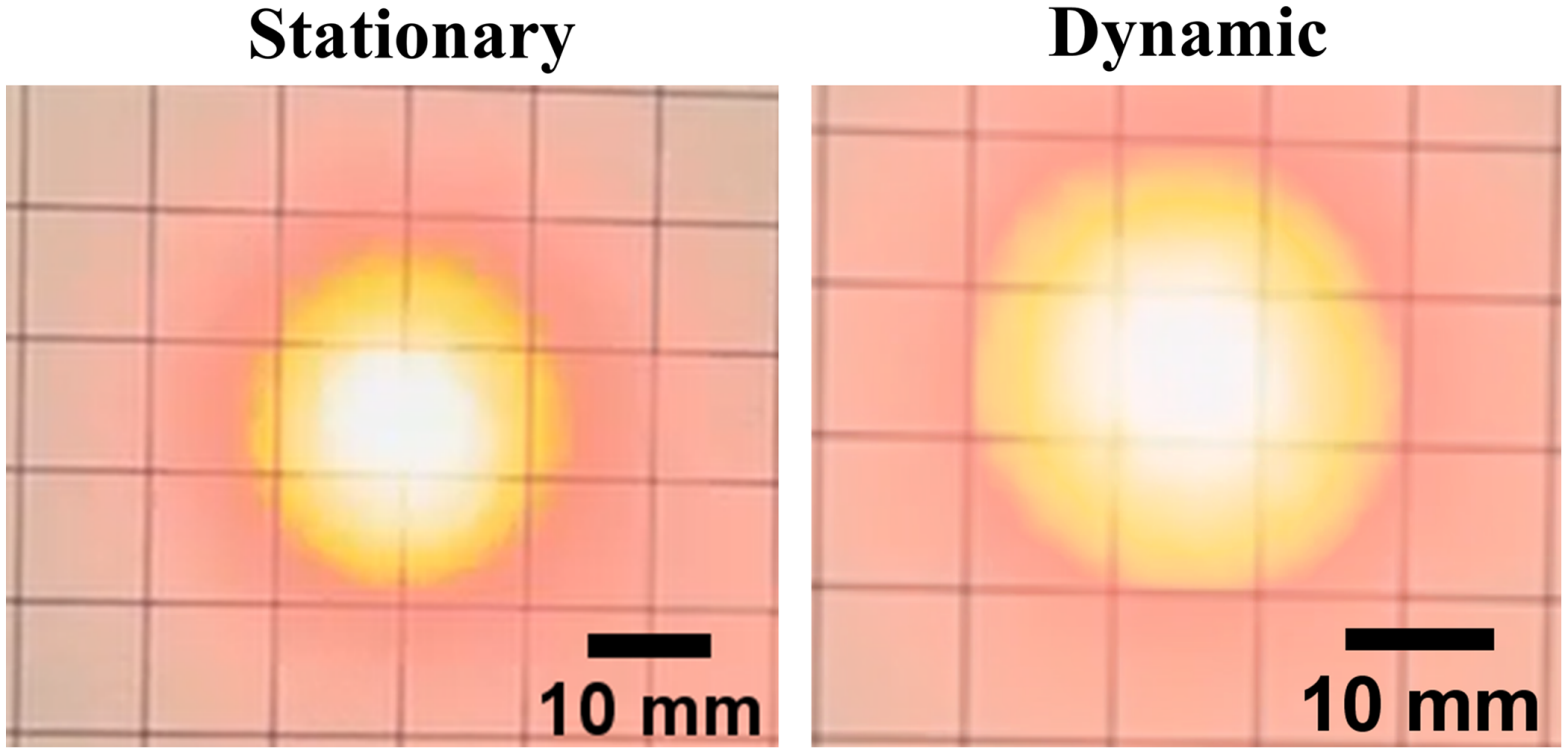
Photographs of beam profiles obtained with stationary and dynamic light delivery.

Figure 7 shows the experimental beam profiles obtained with stationary and dynamic light delivery. The resulting radii of the beam profiles were 12.86 and 14.96 mm, respectively. These values differ from those in Fig. 1(c) [and from the corresponding profile areas calculated from Figs. 6(a) and 6(b)] because they were measured at a distance of 31 mm from the optical fiber bundle, rather than at the tip of the optical fiber bundle. Nonetheless, the dynamic light profile is larger than the stationary light profile, as expected.

### 3.3 Impact of Light Delivery Method on Photoacoustic Image Quality

**Fig. 8:**
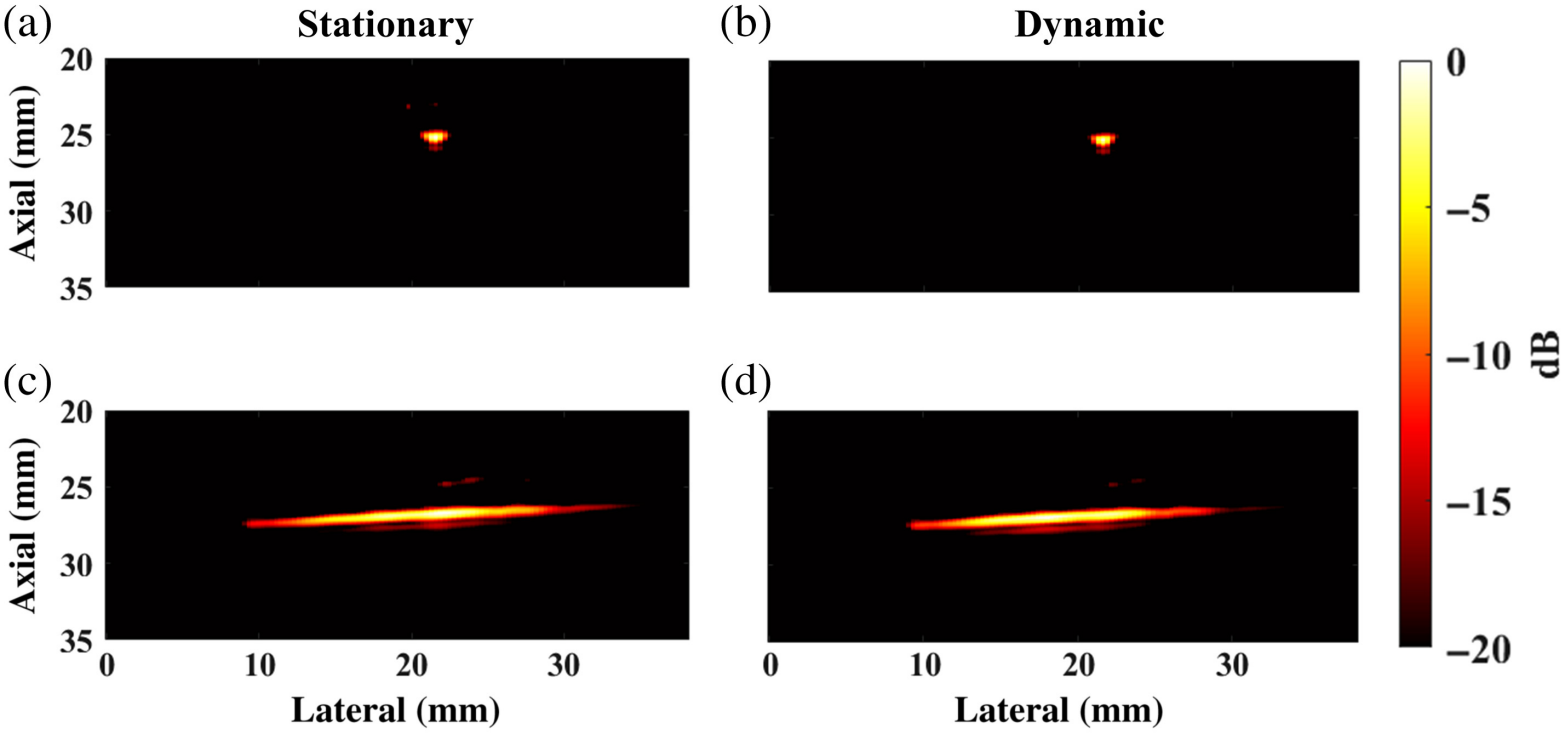
Experimental photoacoustic images of the (a), (b) circular and (c), (d) longitudinal cross sections of a target, acquired with (a), (c) stationary and (b), (d) dynamic light delivery.

Figure 8 shows representative photoacoustic images acquired with stationary and dynamic light delivery for a circular target cross-section and an orthogonal cross-section of the same target positioned at a depth of 26 mm from the transducer. The photoacoustic target is qualitatively detectable in the four images. To quantify detectability in the circular cross-section images, the SNR and gCNR measured 25.63 and 0.95, respectively, with stationary light delivery [Fig. 8(a)] and 25.14 and 0.98, respectively, with dynamic light delivery (Fig. 8(b)).

**Fig. 9:**
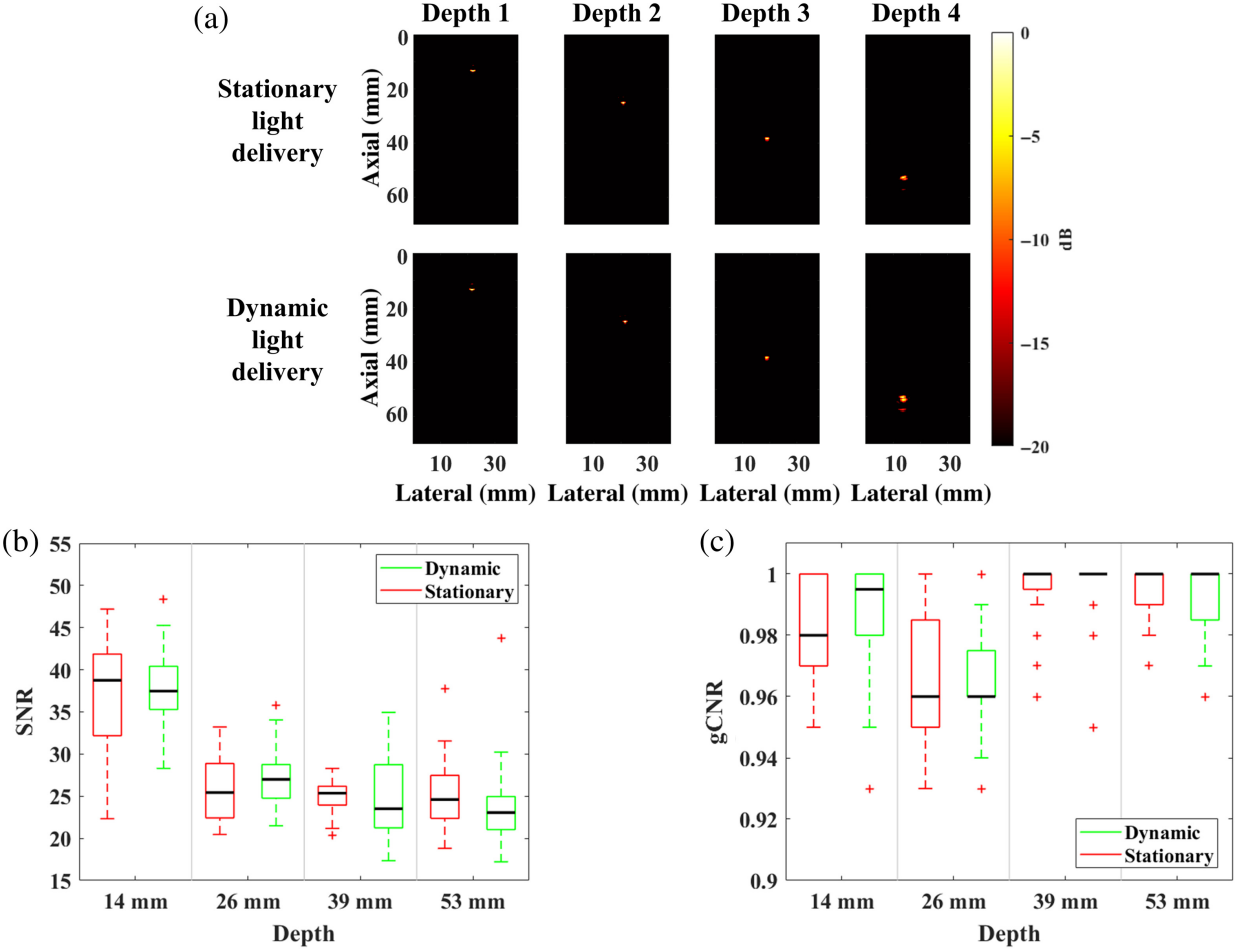
(a) Example photoacoustic images acquired with stationary and dynamic light delivery. Corresponding box-and-whisker plots of the distribution of (b) SNR and (c) gCNR values as a function of target depth.

Figure 9 shows representative photoacoustic images of targets located at four depths from the transducer and corresponding gCNR and SNR distributions from the acquired data as a function of depth and light delivery method (measured from 192 images total per metric). The highest SNR from the representative photoacoustic images was obtained with the shallowest target, which was placed at a depth of 14 mm from the phantom surface (i.e., 38.78 and 37.66 SNR obtained with stationary and dynamic light delivery, respectively). For deeper targets (i.e., 26, 39, and 53 mm depths), SNR values were similar, ranging from 24.63 to 25.54 for stationary light delivery and from 22.86 to 26.66 for dynamic light delivery. The gCNR values remained within the 0.98 to 1.00 range for the acquired images. There were no statistically significant differences between stationary and dynamic light delivery based on SNR (p=0.89) and gCNR (p=0.25) measurements.

**Fig. 10:**
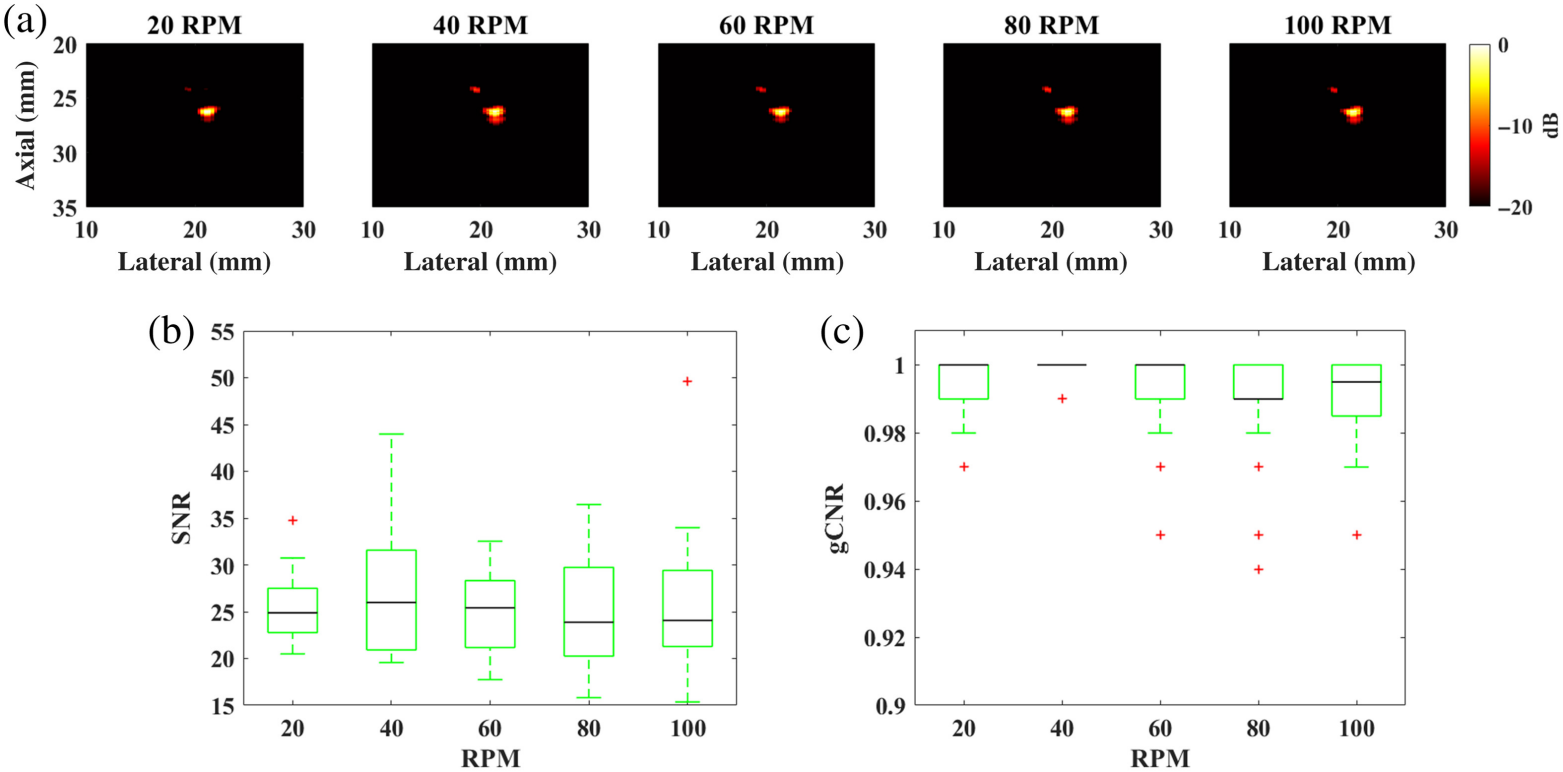
(a) Example photoacoustic images acquired with dynamic light delivery and corresponding distribution of (b) SNR and (c) gCNR values as a function of rotation speed.

Figure 10 shows representative photoacoustic images acquired with dynamic light delivery and the corresponding distribution of SNR and gCNR values as a function of rotation speed (measured from 120 total images per metric). The SNR and gCNR values of the representative images are 24.48 and 1.00, 25.34 and 1.00, 25.76 and 1.00, 25.38 and 0.99, 23.93 and 0.99, respectively, at rotation speeds of 20, 40, 60, 80, and 100 RPM, respectively. The median SNR ranged 23.88 to 25.99, and gCNR remained within the 0.94 to 1.00 range across the five rotation speeds. There were no statistically significant differences between stationary and dynamic light delivery, based on the SNR (p=0.89) and gCNR (p=0.25) measurements. Therefore, rotation speed does not significantly impact the image quality of photoacoustic images acquired with dynamic light delivery.

### 3.4 Histopathology Assessments

**Fig. 11:**
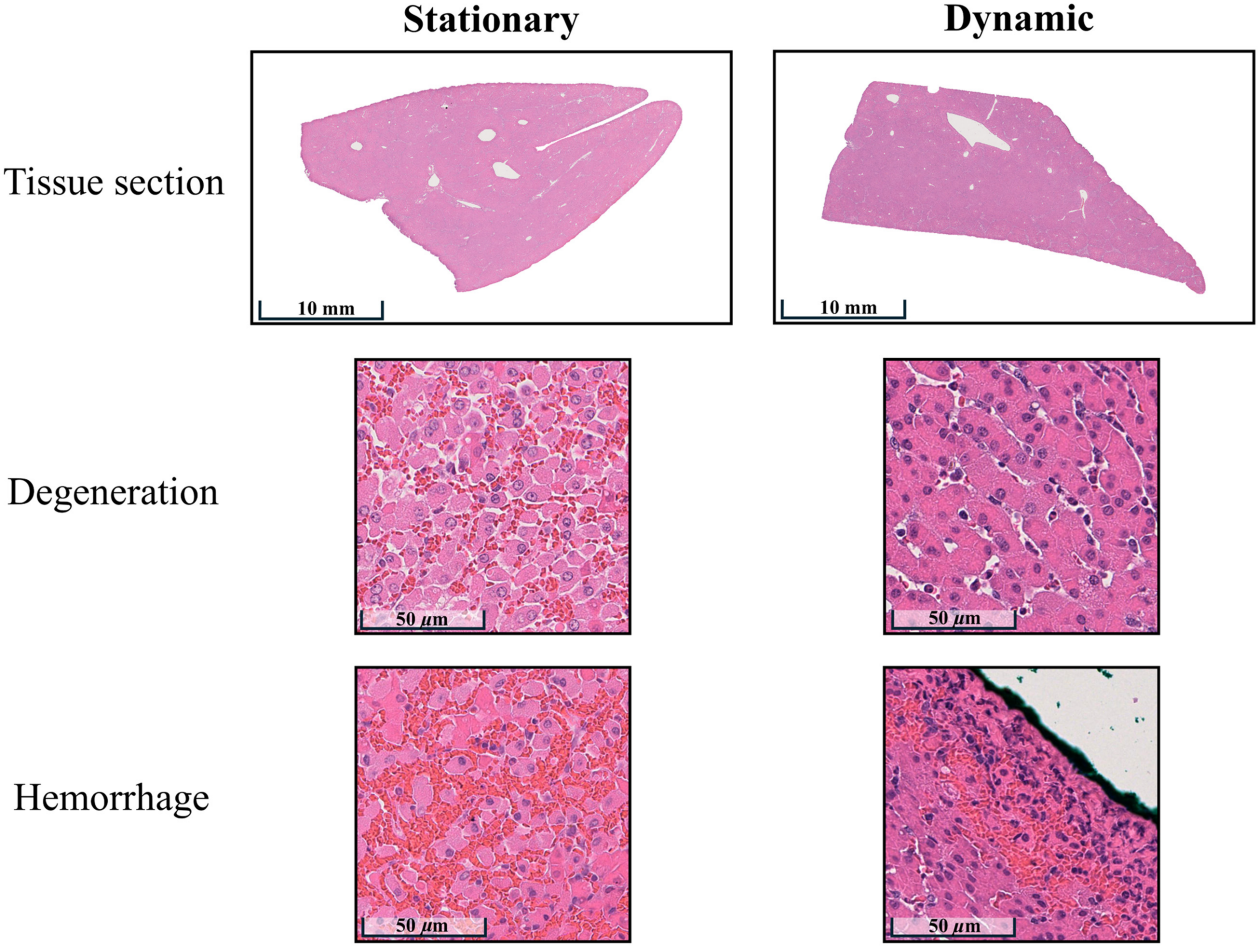
H&E sections from swine liver samples irradiated using stationary and dynamic light delivery. Regions of interest (ROIs) were selected to perform histopathology grading.

Figure 11 shows the pathological grading results of the H&E-stained sections. No swine experienced tissue inflammation or necrosis. The swine sample irradiated using stationary light delivery presented mild hemorrhage and minimal degeneration, whereas the swine sample irradiated using dynamic light delivery showed minimal hemorrhage and absent degeneration. In addition to the hemorrhage grading being greater with stationary light delivery, the area affected by hemorrhage was larger with stationary (rather than dynamic) light delivery.

**Fig. 12:**
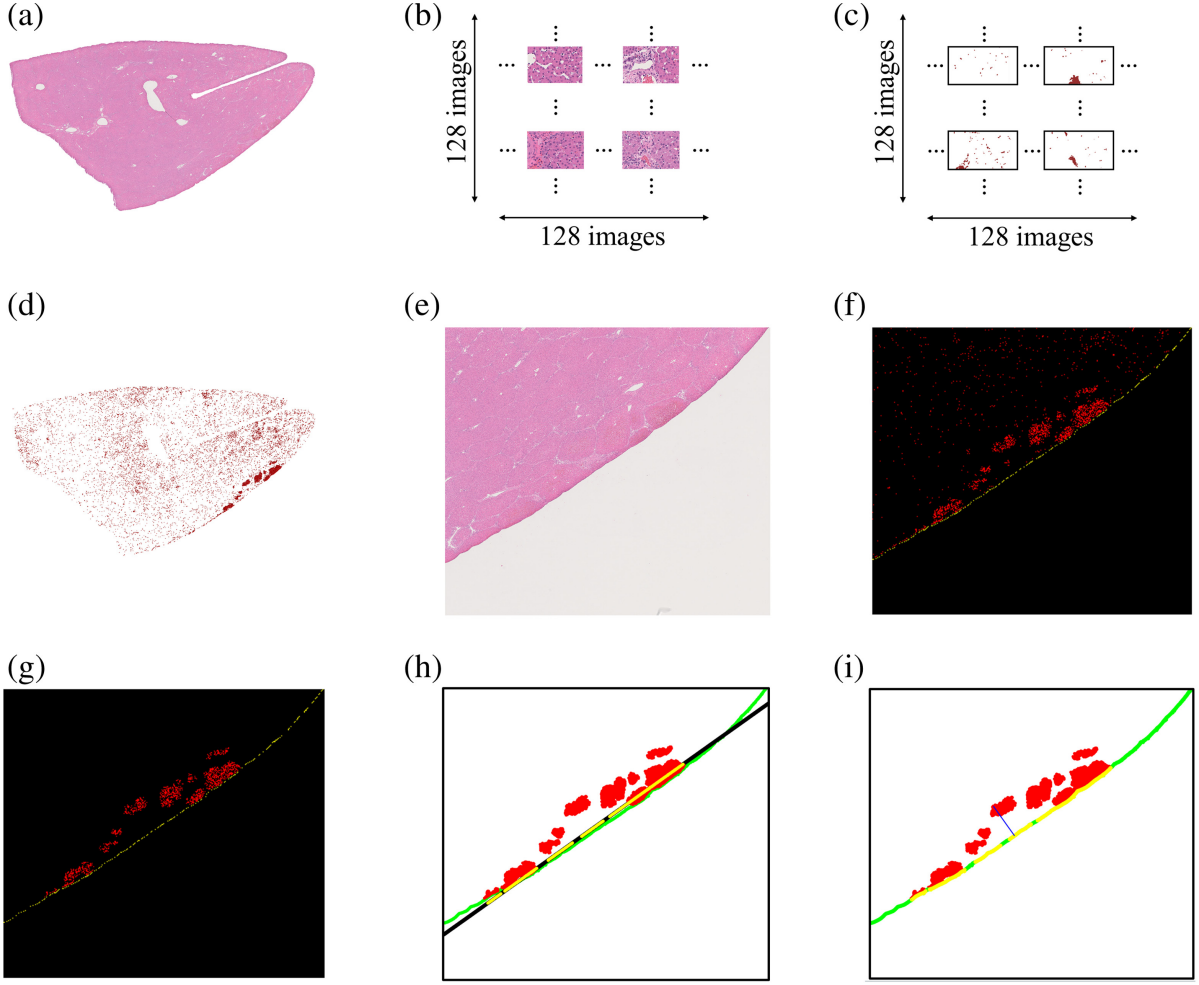
Example outputs of the image processing workflow for hemorrhage depth quantification of H&E sections: (a) H&E section, (b) mosaic of 16,384 images, (c) segmentation (Step 1 in Sec. 2.6.3), (d) red cell segmentation and associated spatial alignment (Step 2), (e) ROI from an H&E section (Step 6), (f) hemorrhage mask (obtained after Step 8), where the red region represents red cells and yellow is the irradiated boundary, (g) filtered hemorrhage mask (Step 9), (h) linear fitting of the green irradiated boundary represented as a black line (obtained from Step 10), where the yellow segments are made of points from the fitted boundary and are used to trace lines between the irradiated boundary and each red cell before distance measurement (Step 11), and (i) blue line representing the normal distance between the irradiated boundary and the farthest red cells (Step 14).

Figure 12 shows example outputs of the image processing workflow for hemorrhage depth quantification of H&E. The H&E section in Fig. 12(a) was transformed into a mosaic of 16,384 images [Fig. 12(b)], followed by segmentation [Fig. 12(c)], red cell segmentation, and associated spatial alignment [Fig. 12(d)]. The ROI selection from the H&E section shown in Fig. 12(e) was then used to ultimately create a hemorrhage mask [Fig. 12(f)], followed by a filtered hemorrhage mask [Fig. 12(g)]. Linear fitting of the irradiated boundary [Fig. 12(h)] ultimately resulted in the blue line shown in Fig. 12(i), which was used to obtain the corresponding hemorrhage depth measurement.

**Fig. 13:**
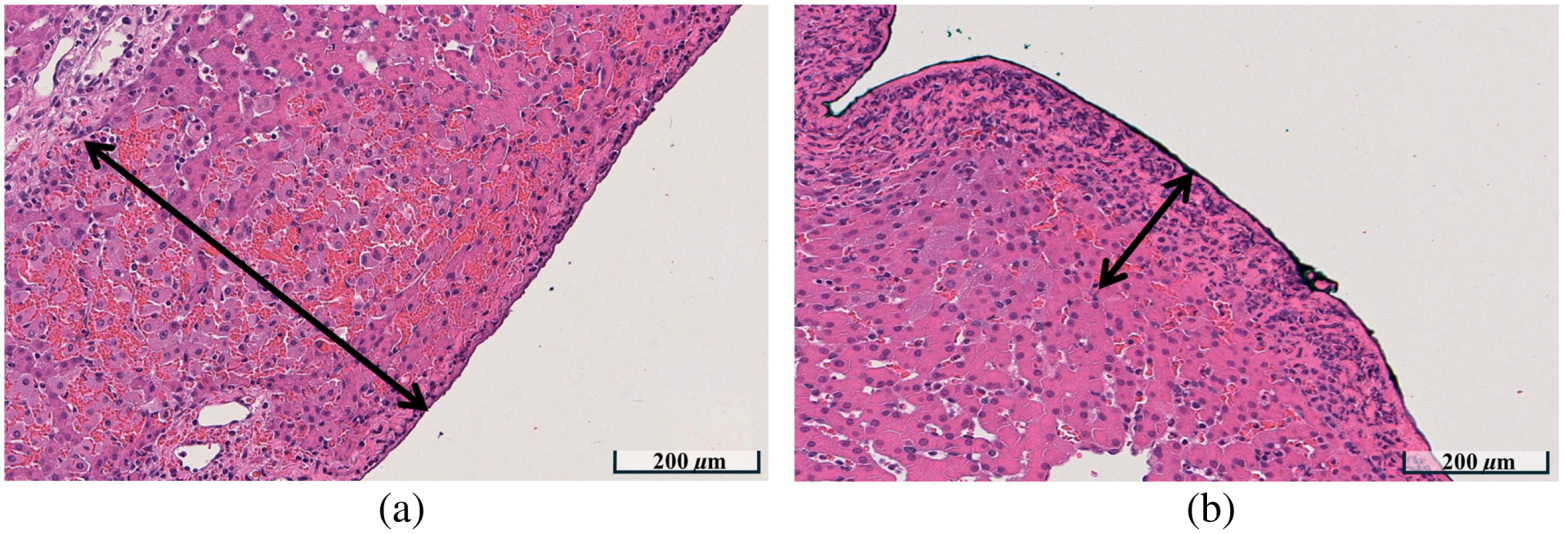
Hemorrhage depth assessment examples on ROIs from H&E sections corresponding to (a) stationary light delivery and (b) dynamic light delivery. The distance between the laser-exposed boundary and the deepest laser-induced hemorrhage cluster was represented by black arrows in each case. These arrows visually emphasize the difference in hemorrhage depth.

Figure 13 shows annotated H&E sections from the resected swine liver specimens irradiated using stationary [Fig. 13(a)] and dynamic [Fig. 13(b)] light delivery to demonstrate representative hemorrhage depth measurements. The annotations indicate the deepest hemorrhage cluster relative to the irradiated tissue boundary within the ROI depicted, with the length of each arrow indicating the maximum depth of hemorrhage within each ROI. A greater number of hemorrhage clusters were observed at deeper depths with stationary light delivery [Fig. 13(a)] compared with dynamic light delivery [Fig. 13(b)]. The overall DSC, IoU, recall, and precision of the Attention U-Net when segmenting red cells from individual JPEG images such as these were 0.99, 0.99, 0.99, and 0.99, respectively.

**Fig. 14:**
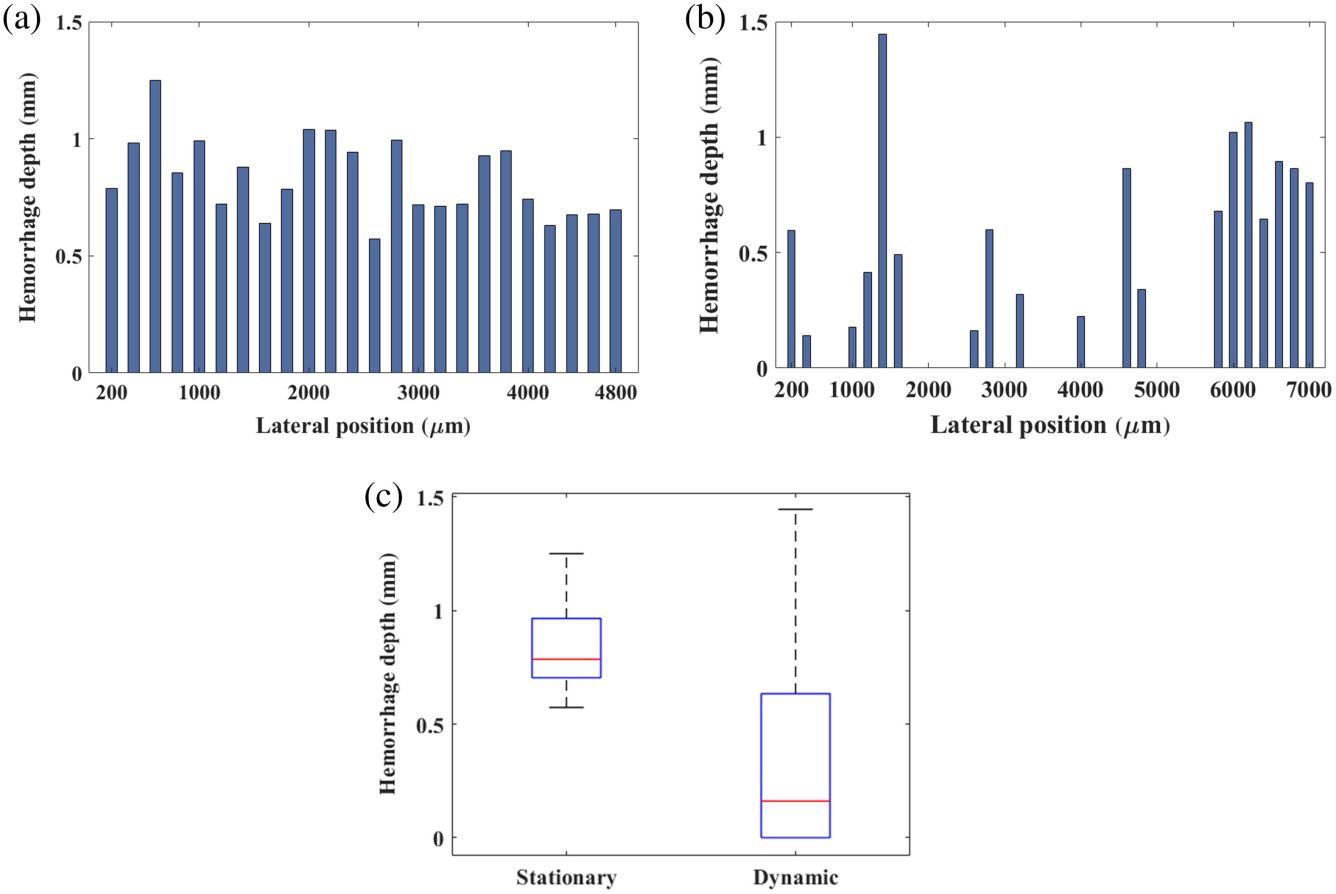
Hemorrhage depth quantification for individual H&E sections corresponding to (a) stationary light delivery, (b) dynamic light delivery, and (c) distribution of hemorrhage depth measurements shown as box-and-whisker plots. The median energy is indicated by the red horizontal line, the interquartile range is indicated by the top and bottom bounds of each box, and the maximum and minimum values are indicated by the lines extending from each box. A larger number of H&E sections were used for dynamic light delivery as this approach illuminates a larger surface area than stationary light delivery.

Figure 14 quantifies the maximum hemorrhage depth achieved with stationary and dynamic light delivery as a function of lateral position along the tissue surface. Dynamic light delivery [Fig. 14(b)] produces a greater number of lateral positions than stationary light delivery [Fig. 14(a)], which is expected as the dynamic approach covers more tissue area than the stationary approach. Figure 14(c) summarizes these results with box plots showing the median and interquartile range of hemorrhage depth measurements as 0.79 and 0.25, respectively, with stationary light delivery and 0.16 and 0.62, respectively, with dynamic light delivery. The spike at the lateral position of 1400  μm in Fig. 14(b) is responsible for the maximum value and large interquartile range shown in Fig. 14(c). This spike occurs because the empirical threshold value intended to exclude non-hemorrhage-related clusters (i.e., Step 9 of Sec. 2.6.3) unintentionally preserved red cells from a small vessel, yielding an inaccurate value. Nonetheless, the median hemorrhage depths reduced from 0.79 mm with dynamic light delivery to 0.16 mm with stationary light delivery, resulting in an 80% hemorrhage depth reduction ($p=8.59\times 10^{-6}$).

## 4 Discussion

This study is the first to introduce a motion-based, dynamic light delivery method, which is designed to mitigate adverse thermal effects during photoacoustic imaging. Monte Carlo simulations predicted 1.85 lower energy densities at the tissue surface with dynamic rather than stationary light delivery (Fig. 6). Experimentally, dynamic light delivery qualitatively reduced the hemorrhage categorization from mild to minimal (Figs. 11 and 13) and reduced the median laser-related hemorrhage depths by 80%, when compared with stationary light delivery (Fig. 14). It is additionally beneficial that there were no statistically significant differences between the image quality obtained with our proposed approach and with a more conventional stationary light delivery approach (Figs. 9 and 10). These results are promising to address potential laser safety concerns associated with photoacoustic imaging and thereby advance clinical translation.

When evaluating the effectiveness of dynamic light delivery to reduce laser-related thermal damage, the results herein (and our previous report^25^) focus on the associated radius as the primary parameter to describe the trajectory and analyze the associated local energy density distribution. Another possible design parameter is rotation speed. When applied in a linear trajectory, increasing the laser speed is expected to spread the associated laser energy over a larger surface area, thereby delivering lower local energy^37^ and a lower degree of thermal damage (given the insufficient time to exchange heat between the tissue and the laser^38^). However, when a light source is applied in circular trajectories, an apparent paradox occurs as both low and high laser speeds can lead to thermal damage. A lower rotation speed leads to longer laser exposure times in a given region, which can potentially induce thermal damage. A higher rotation speed translates to a shorter motion period which promotes frequent heating of a particular region and can cause heat build-up over time. Conversely, numerical analyses suggest that a higher rotation speed leads to a lower temperature peak.^39^ Overall, adjusting the rotation speed of our laser positioning device within the range of values we investigated could potentially reduce laser-related thermal damage, without impacting image quality (Fig. 10).

In contrast to the alternative option of implementing cooling devices,^19^^–^^22^^,^^24^ our laser positioning device is customizable, as it can be tailored to accommodate light sources of varying shapes and sizes, to operate with motors of different shapes and sizes, and to provide trajectories of different radii than the 1.5-mm radius used in our study, making it suitable for a wide range of setups. In addition, the laser positioning device is potentially compatible with different laser systems, as its design is independent of the characteristics of the laser system. Future work will expand our experimental methods to include variable design parameters and other internal and external organs.

The required rotation of a light source for dynamic light delivery can be considered an inconvenience due to the following reasons. First, it is simpler to fix (or minimally vary) any handheld light delivery mechanism. However, our implementation ensures controlled motion to avoid energy “hotspots.” Second, maintaining proper and continuous contact between the light source and the tissue during rotation is necessary, as misalignment compromises energy transfer. Therefore, we designed a housing that stabilizes the components and maintains a light source position perpendicular to and in direct contact with the imaged tissue. Third, the bulkiness of our design may be considered an additional inconvenience. To overcome this inconvenience in the future, a smaller optical fiber bundle can potentially be employed, which would enable the integration of a smaller motor with lower torque requirements. The motor could also be repositioned to reduce the footprint of the device. In particular, relocating the motor higher than the gears would allow for only the optical fiber to contact the tissue, further minimizing the footprint of the device.

One potential application of dynamic light delivery is photoacoustic breast imaging in individuals with darker skin tones.^40^ As skin tone darkens, reduced light fluence and increased acoustic clutter can introduce bias in photoacoustic imaging.^41^ Integrating our approach into breast photoacoustic imaging could potentially enable elevated laser energies while mitigating thermal damage. Considering the common design of compactly integrating a handheld ultrasound transducer with light sources, motors can potentially be attached to light sources housed within a probe to achieve the desired application. Surgical guidance^4^^,^^42^ with this design (or with the setup shown in Fig. 5) is another potential application.

One possible limitation of our current dynamic light delivery design is the requirement for a $3011.94\;{\mathrm{mm}}^{2}$ footprint, as opposed to the $19.64\;{\mathrm{mm}}^{2}$ footprint with stationary light delivery. Although the larger footprint may be challenging for smaller surfaces, this footprint is considered acceptable for larger surface structures (e.g., skin). Therefore, the effectiveness of dynamic light delivery depends on the specific anatomical structures being imaged. It is also possible to introduce more compact designs based on the same concepts presented herein to optimize utility for multiple clinical or surgical applications. While a diverging lens placed at the tip of the optical fiber could potentially expand the illuminated area, a larger, uniform, stationary illumination would reduce the local energy fluence. Instead, our dynamic illumination method displaces the light source over time, preserving the original energy fluence of the light source without compromising photoacoustic image quality.

## 5 Conclusion

The work presented herein is the first to introduce controlled, dynamic light delivery as an innovative approach to minimize potential laser-related thermal damage during photoacoustic imaging. The proposed device takes advantage of continuous and predictable laser source displacements without requiring operator intervention during imaging. Based on qualitative pathological readings, quantitative hemorrhage depth assessment, and image quality metrics, this dynamic light delivery approach effectively minimizes thermal damage without compromising the image quality achievable with conventional stationary light delivery methods. The presented approach and its associated outcomes have the potential to address laser safety in photoacoustic-based diagnostic imaging and in photoacoustic-guided surgery, as well as in other optics-based diagnostic or surgical applications that are intended to be safe.

## Data Availability

The code and data used in this study can be made available upon reasonable request to the corresponding (JA) and senior (MALB) authors.
